# Factors associated with health-related quality of life in survivors of childhood-onset and adult-onset cancer compared to controls

**DOI:** 10.1007/s00520-026-10775-y

**Published:** 2026-05-14

**Authors:** Madeleine Rhodes, Christina Signorelli, Mark W. Donoghoe, Jordana K. McLoone, Claire E. Wakefield, Antoinette Anazodo, Ursula M. Sansom-Daly, Anne-Sophie Darlington, Samantha C. Sodergren, Richard J. Cohn, Joanna E. Fardell

**Affiliations:** 1 Kids Cancer Centre, Sydney Children’s Hospital, Randwick, Australia; 2 Behavioural Sciences Unit, School of Clinical Medicine, Discipline of Paediatrics and Child Health, Randwick Clinical Campus, UNSW Medicine & Health, Sydney, UNSW Australia; 3 Clinical Research Unit, UNSW Medicine & Health, Sydney, UNSW Australia; 4 Division of Quality of Life and Pediatric Palliative Care, Department of Pediatrics, Stanford University and Stanford Medicine Children’s Health, Palo Alto, CA USA; 5 Sydney Youth Cancer Service, Nelune Comprehensive Cancer Centre, Prince of Wales Hospital, Randwick, Australia; 6 School of Health Sciences, University of Southampton, Southampton, UK; 7 Western Sydney Youth Cancer Service, Westmead Hospital, Sydney, Australia

**Keywords:** Health-related quality of life, Childhood cancer, Adult cancer, Oncology, Survivorship, Controls

## Abstract

**Purpose:**

This cross-sectional survey study explored health-related quality of life (HRQoL) across adult survivors of childhood- or adult-onset cancer in Australia, compared to controls with no history of cancer, and assessed factors associated with HRQoL among cancer survivors.

**Methods:**

Participants completed a survey including clinical and demographic factors and HRQoL (assessed using EQ-5D-5L).

**Results:**

Childhood cancer survivors (< 16 years at diagnosis, *n* = 403) and adult-onset cancer survivors (> 16 years at diagnosis, *n* = 656) participated, alongside 901 controls. Overall HRQoL was comparable between childhood cancer survivors and controls, whilst adult cancer survivors reported better HRQoL than controls. Over 50% of child and adult cancer survivors reported meaningful reductions in overall HRQoL compared to perfect health (i.e. EQ-5D-5L index < 0.92). Childhood survivors reported more mobility and activity limitations on the EQ-5D-5L domains compared to controls, whilst adult survivors reported fewer self-care issues. Childhood survivors' poorer HRQoL was significantly associated with cancer diagnosis and treatment, greater health issues, lower resilience, and smoking history. Adult survivors' poorer HRQoL was associated with older age, lower education, fewer health issues, lower resilience, and less physical activity.

**Conclusion:**

This study provides a comprehensive examination of HRQoL among Australian cancer survivors, shedding light on age-specific differences and the multifaceted nature of associated factors.

**Implications for cancer survivors:**

Factors associated with HRQoL highlight modifiable opportunities for targeted survivorship care interventions that may improve survivors’ long-term coping and adaptation, as well as broader physical health after cancer.

**Supplementary Information:**

The online version contains supplementary material available at 10.1007/s00520-026-10775-y.

## Introduction

Advances in cancer treatment have led to greater survival rates, with approximately 80% of childhood cancer patients and 50% of adult-onset cancer patients surviving five or more years post-treatment in high-income countries [1, 2]. However, survivors are at a heightened risk of developing lasting treatment-related morbidity including second malignancies and cardiovascular, endocrine, gastrointestinal, immunologic, musculoskeletal, neurologic, and pulmonary morbidities [3, 4]. These effects are compounded by the financial burden of life-long healthcare costs [5]. The chronic health implications of cancer can adversely impact survivors’ health-related quality of life (HRQoL). HRQoL is the subjective appraisal of life in relation to physical, social, and psychological health circumstances [6]. Research consistently highlights that HRQoL is one of the primary concerns and priorities for people living with and beyond cancer [7, 8].

Patient-reported HRQoL indicators are becoming increasingly important in understanding survivor wellbeing and informing effective survivorship care. Previous studies have established that overall HRQoL diminishes during treatment and improves soon after treatment completion in survivors of both childhood and adult cancer [6, 9, 10]. However, results are inconclusive when comparing HRQoL of these survivor cohorts to population controls. Survivors of childhood cancer (aged < 18 years at diagnosis) have been reported to experience overall HRQoL that is worse [11, 12], similar [13, 14], or better [15, 16] than age-matched controls. Several studies have shown these survivors experience decreased functioning within specific HRQoL domains, including psychological wellbeing, physical and social health [13, 17]. There is mixed evidence on the symptoms predictive of HRQoL among survivors of adult-onset cancers (aged > 18 years at diagnosis) compared to matched controls [6, 18, 19], and population averages [20, 21] although poorer outcomes across multiple domains of quality of life have been observed among adult survivors such as mobility [22], cognition [23, 24], anxiety and depression [25], and ability to perform usual activities [26].

Poorer HRQoL outcomes in childhood cancer survivors when they reach adulthood are associated with female sex, being unpartnered, older age, smoking, and low physical activity levels and more intensive treatments such as cranial radiation [27–30]. For adult-onset cancer survivors receiving chemotherapy treatment [25], having a greater number of comorbid health conditions [31], smoking [32], and reporting a greater fear about cancer recurrence [33, 34], have been associated with decreased HRQoL, as well as female sex and low physical activity levels [18]. In addition, given that breast cancer remains the most common cancer diagnosis among women, with high 5-year survival rates (on average 91%), this group remains of interest in adult cancer survivor HRQoL research particularly relating to investigating factors associated with HRQoL [35].

As is the case globally, in Australia and New Zealand survivorship care varies dramatically according to age at diagnosis and the availability of appropriate survivorship care, for example, survivors of childhood cancer can be lost to follow-up in transition across paediatric to adult services, despite increasing risk of late effects [36–38]. Unsurprisingly, the nature of survivorship care can markedly impact survivors later HRQoL in different ways [39]. However, few studies have presented a complete picture of HRQoL across the lifespan (i.e. both childhood and adult-onset survivor cohorts) or in Australian and New Zealand survivors, and existing evidence is mixed.

Therefore, this study aimed to 1) assess Australian and New Zealand cancer survivors’ HRQoL by age at diagnosis (childhood or adulthood) and compared to those without a history of cancer, and 2) investigate factors associated with HRQoL including lifestyle behaviours such as history of smoking and physical activity, and total number of reported health issues experienced following cancer treatment. These predictors were selected based on prior evidence that lifestyle behaviours (smoking, physical activity) and the cumulative burden of cancer-related health issues are strongly associated with HRQoL – as described above—allowing us to examine modifiable and clinically relevant factors across survivorship. We analyze data across childhood and adult-onset survivors separately, to strengthen our understanding of novel shared individual and lifestyle factors that are most strongly associated with HRQoL among cancer survivors, and across the lifespan, which may be critical to inform a holistic approach to survivors’ care.

## Methods

Ethics approval was granted by all participating hospitals (HREC/12/POWH/345) and UNSW Sydney (HC15773). This study was endorsed by the Australian and New Zealand Children’s Haematology Oncology Group (ANZCHOG), and is a secondary analysis from the ANZCHOG Survivorship Study survey (detailed methods reported elsewhere) [40]. The childhood cancer survey was developed through a multi-stage process in collaboration with lead experts in cancer survivorship across ANZCHOG centres across Australia and New Zealand, pilot tested and iteratively improved, and then adapted through further consultation with physicians treating in adult (> 18 years) oncology centres in Australia to expand recruitment to include adult-onset and control populations.

### Participants and recruitment

Eligible survivors were ≥ 18 years old at the time of participation, had received treatment for cancer at a hospital in Australia (childhood cancer and adult-onset cancer survivors) or New Zealand (childhood cancer survivors only), were in remission, and proficient in English.

We recruited survivors of childhood cancer who had been diagnosed at ≤ 18 years old and were ≥ 5 years post active treatment We used electronic hospital records from 11 participating hospitals to identify potential participants. 1855 childhood cancer survivors were invited to participate. We posted or emailed an invitation to participate, signed by the potential participant’s treating oncologist, and provided a study pack which included a consent form, information sheet, and questionnaire. We included a reply-paid envelope and card with a web-link to participate online if preferred. We followed up non-respondents up to four times via phone call.

Survivors of adult-onset cancer (diagnosed when they were older than eighteen years) were recruited via the Register4 online database, which has > 40,000 members who are primarily female (96.6%, age range 20–96) as Register4 was started with breast cancer patients and subsequently expanded to survivors of any cancer [41]. Survivors were eligible if they had completed cancer treatment at least six months prior to participating in the study. Register4 advertised our study on their website and members were emailed a generic invitation letter and study pack. Non-respondents received one follow-up email.

Controls were community participants who had opted-in to participate via an online survey community; PureProfile Pty. Ltd. PureProfile is a database of Australians, primarily engaged through online surveys and radio advertisements. Survey respondents receive small payments for their participation (approximately $5AUD). Controls were approximately matched to survivors by age and state of residence. We excluded respondents in this group who were cancer survivors or had cared for a child with cancer.

### Data collection

#### Clinical and demographic measures

All participants self-reported socio-demographic characteristics including age, gender, education level, employment status (employed or unemployed), marital status (married/de facto or not married/de facto) and location. We coded location into major city (metropolitan) or rural/regional groups using the Accessibility Remoteness Index of Australia (ARIA) classification for Australian participants and the New Zealand Area Codebook for New Zealand participants. Survivors reported clinical characteristics including cancer diagnosis, treatment/s received (chemotherapy, radiotherapy, surgery and/or bone marrow transplantation), and date/s of cancer diagnosis, treatment completion and of relapse if relevant.

#### Health-related quality of life measure

All participants completed the EQ-5D-5L, a validated tool commonly used in cancer survivors which assesses health-related quality of life overall and health status within five specific domains (mobility, self-care, ability to participate in usual activities, pain/discomfort, and anxiety/depression) [42, 43]. Domains are self-reported on a 5-point scale (‘no problems’ to ‘I am unable to’), which we reported as the proportion of participants reporting any domain-specific difficulties and also used to determine a quality-of-life index value ranging from 0 (death) to 1 (perfect health), according to developer guides [44].

#### Covariates

Among all survivors, we asked participants to report on lifestyle factors including their physical activity level (minutes spent active per week, dichotomised as insufficient and sufficient according to guidelines)[45] and smoking status (‘never smoked’, vs ‘ex-smoker’/‘current smoker’). We also measured late effects using a purpose-designed question in which survivors indicated on a list of commonly experienced cancer-related health issues (e.g. pain, lung problems, fertility issues) that they thought they had experienced or were experiencing since finishing cancer treatment. These were derived from a literature search of commonly reported health problems and summed to derive the total number of health issues survivors had/were experiencing. We measured survivor resilience using the two-item Connor–Davidson Resilience Scale (CD-RISC2) which asks participants to indicate whether they are ‘Able to adapt to change’ and ‘Tend to bounce back after hardship or illness’ [46]. Scores were summed, with higher scores indicating higher resilience.

#### Data analysis

Statistical analysis was conducted using R version 4.3.1 [47], with the mgcv package used to fit models [48], gratia [49] and ecostats [50] to perform parametric bootstrapping, and ggplot2 to visualise model fits [51]. Noting each cohort recruited has different inclusion criteria (> 5 years post-treatment for childhood cancer survivors, > 6 months for adult-onset cancer survivors), we reported results separately within each group to characterise outcomes across the lifespan, rather than directly comparing them.

We identified survivors with a minimally important, or clinically meaningful, reduction in HRQoL compared to perfect health as those reporting an overall index value less than 0.92, consistent with MID estimates published for the EQ-5D in cross-sectional studies [52, 53]. To assess participants’ HRQoL, for each EQ-5D-5L domain we fitted a binary logistic generalised additive model to estimate the association between age and the probability of reporting any problems, separately within each group (childhood cancer survivors, adult-onset cancer survivors and controls). We used generalised likelihood ratio tests to test for a difference in these associations between controls and each group of survivors. Overall EQ-5D-5L index scores were also analysed using this approach, but with the index score “deficit” (one minus the index score) modelled using a Tweedie distribution and log link, which allows for the fact that many participants had the maximum index score of one (a deficit of zero). Exponentiated coefficients from the models can be interpreted as the estimated relative change in the index score deficit (the “relative deficit”) associated with each variable, where values larger than one indicate a greater deficit, and hence lower HRQoL.

To investigate factors associated with HRQoL, the index score deficit was modelled using generalised additive models with a Tweedie distribution separately within each survivor group. Pre-specified factors of interest (the clinical, demographic, and covariates listed above) informed by the literature were included as explanatory variables in univariable and multivariable models [27–32], with continuous variables fitted using smooth terms. In adult-onset survivors, sex and diagnosis were mutually adjusted in multivariable models to reduce confounding in a sample predominantly comprising women with breast cancer, such that sex reflects a comparison between men and women without breast cancer and diagnosis reflects breast versus non-breast cancer among women. Because cancer treatment is heavily dependent on diagnosis, the models included an interaction between these variables. Due to the potentially complex interplay between the effects of current age, time since diagnosis and age at diagnosis, the model for adult-onset survivors also included a smooth two-dimensional interaction between current age and time since diagnosis. P-values to test for the effect of each variable in each model were calculated using a likelihood ratio test based on a parametric bootstrap sample.

## Results

We recruited 1,059 eligible cancer survivors. Childhood cancer survivors (*n* = 403, 54% response rate, 56.3% female) were on average twenty-six years old (range 16–61), 19.0 years since diagnosis (range 5–59 years), and had most commonly been treated for leukaemia (*n* = 174, 43%). Adult cancer survivors (*n* = 656, response rate unknown due to recruitment method, 94.7% female) were on average sixty-one years old (range 27–83), 9.4 years since diagnosis (range 0–54 years), and had most commonly been treated for breast cancer (*n* = 439, 67%). Controls (*n* = 901) were on average fifty years old (range 15–100). Further demographic data are summarised in Table 1.

**Table 1:** Participants characteristics table for survivors and controls

Outcome	Childhood cancer survivors [*N* = 403]	Adult-onset cancer survivors [*N* = 656]		Controls [*N* = 901]
Age, years^a^*(mean* ± *SD)*	26.2 ± 7.6	61.2 ± 9.9		50.0 ± 17.6
Range	16–61	27–83		15–100
Sex^b^*n (% female)*	227 (56.3%)	621 (94.7%)		671 (74.5%)
Education^c^*n (%)*				
Secondary	185 (45.9%)	111 (16.9%)		311 (34.5%)
Tertiary	208 (51.6%)	545 (83.1%)		590 (65.5%)
Employment^d^*n (%)*				
Employed	270 (67.0%)	307 (46.8%)		469 (52.1%)
Unemployed	125 (31.0%)	349 (53.2%)		432 (47.9%)
Income^e^*n (%)*				
< $AUD 60,000	213 (52.9%)	246 (37.5%)		402 (44.6%)
> $AUD 60,000	120 (29.8%)	248 (37.8%)		339 (37.6%)
Prefer not to say	61 (15.1%)	162 (24.7%)		158 (17.5%)
Marital Status^f^*n (%)*				
Married or de facto	98 (24.3%)	473 (72.1%)		493 (54.7%)
Not married or de facto	301 (74.7%)	183 (27.9%)		408 (45.3%)
Place of residence (ARIA class)^g^ *n *				
*(%)*	281 (69.7%)	476 (72.6%)		683 (75.8%)
Metro Regional/Remote	83 (20.6%)	176 (26.8%)		214 (23.8%)
Diagnosis^h^ *n (%)*				
`Lymphoma	57 (14.1%)	11 (1.7%)		-
Leukaemia	174 (43.2%)	7 (1.2%)		-
Brain Cancer	53 (13.2%)	2 (0.3%)		-
Breast Cancer	-	439 (66.9%)		-
Bowel Cancer	-	25 (3.8%)		-
Melanoma	-	22 (3.4%)		-
Uterine	-	17 (2.6%)		-
Prostate	-	14 (2.1%)		-
Other	117 (29.0%)^**i**^	111 (16.8%)^j^		-
Treatment^k^ *n (%)*				
Surgery	184 (45.7%)		639 (97.4%)	-
Chemotherapy	361 (89.6%)		354 (54.0%)	-
Radiotherapy	190 (47.1%)		416 (63.4%)	-
Bone Marrow Transplant	70 (17.4%)		11 (1.7%)	-
Health issues^l^				-
Mean (SD)	3.80 (3.56)		5.56 (3.31)	-
Median [Q1, Q3]	3.00 [1.00, 5.00]		5.00 [3.00, 8.00]	-
Min, Max	0, 19.0		0, 19.0	-
Smoking status^m^				
Never smoked	349 (86.6%)	453 (69.1%)		-
Ever smoked	52 (94.8%)	203 (30.9%)		-

^***a***^ Missing data for survivor age (*n* = 8) and control age (*n* = 12)

^***b***^ Missing data for survivor sex (*n* = 2)

^***c***^ Missing data for survivor education status (*n* = 10)

^***d***^ Missing data for survivor employment status (*n* = 8)

^***e***^ Missing data for survivor income (*n* = 9) and control income (*n* = 2)

^***f***^ Missing data for survivor martial status (*n* = 4)

^***g***^ Missing data for survivor place of residence (*n* = 43) and control place of residence (*n* = 4)

^***h***^ Missing data for childhood cancer diagnosis (*n* = 2) and adult cancer diagnosis (*n* = 8)

^***i***^ For childhood cancer survivors, “other” refers to diagnoses other than lymphoma, leukaemia or brain cancers, and includes Wilms’ tumor, sarcoma, neuroblastoma, hepatoblastoma, followed by retinoblastoma, and germ cell tumors

^***j***^ For adult-onset cancer survivors, “other” refers to unlisted cancer diagnoses (e.g., [non-exhaustive] testicular, renal, bladder, ovarian, cervical)

^***k***^ Missing treatment data for cancer survivors were grouped with “no” responses

^l^ Missing data for childhood cancer survivors’ health issues (*n* = 15)

^m^ Missing data for childhood cancer survivors’ smoking status (*n* = 2)

### Comparisons of HRQoL between cancer survivors and controls

The comparisons of HRQoL included 1,939 participants (394 childhood cancer survivors, 656 adult cancer survivors and 889 controls) whose age was known and who completed at least one question from the EQ-5D-5L questionnaire.

**Fig. 1:**
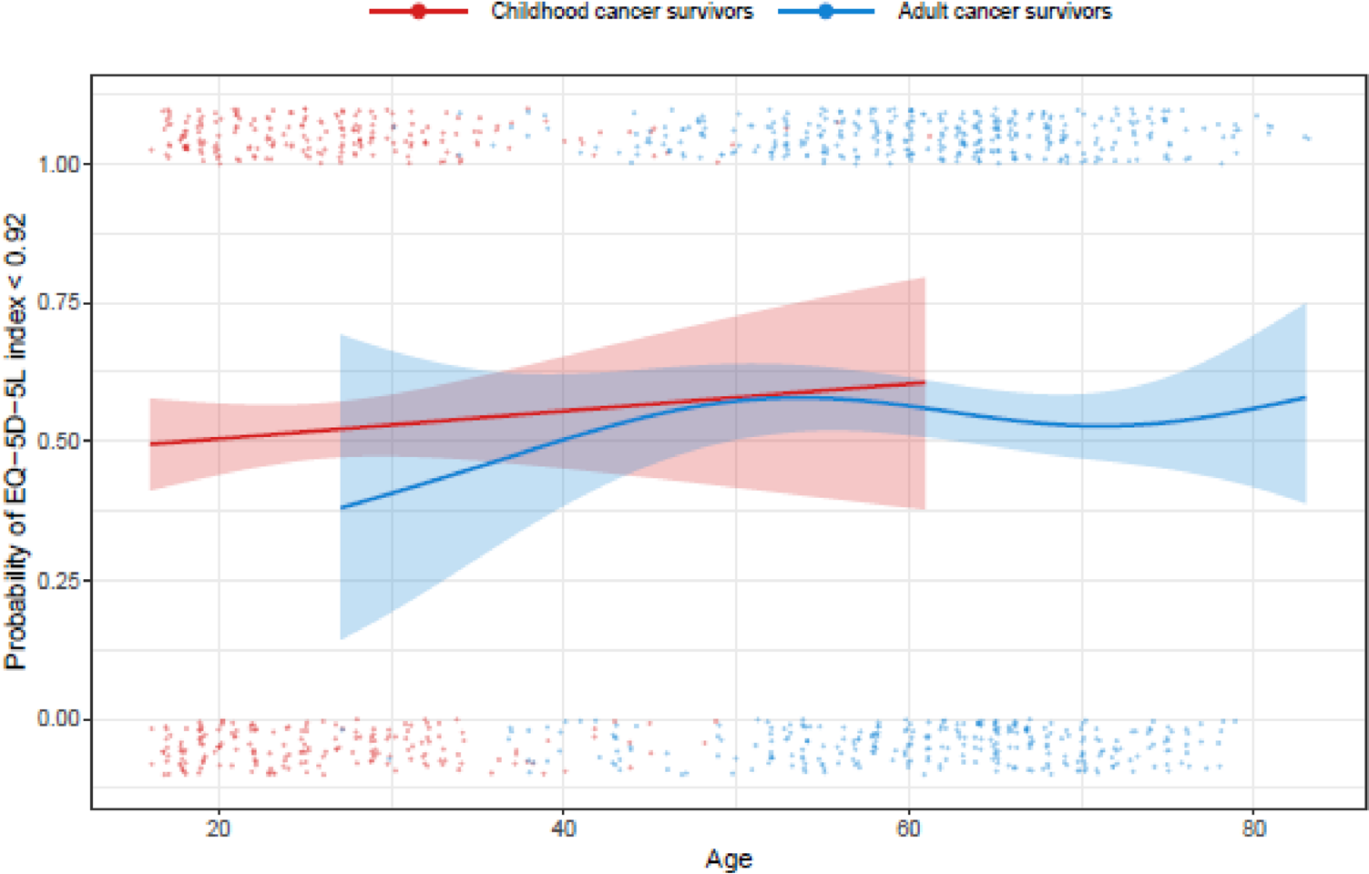
Probability of childhood cancer survivors and adult-onset cancer survivors reporting less than perfect health (i.e. HRQoL index < 0.92)

**Fig. 2:**
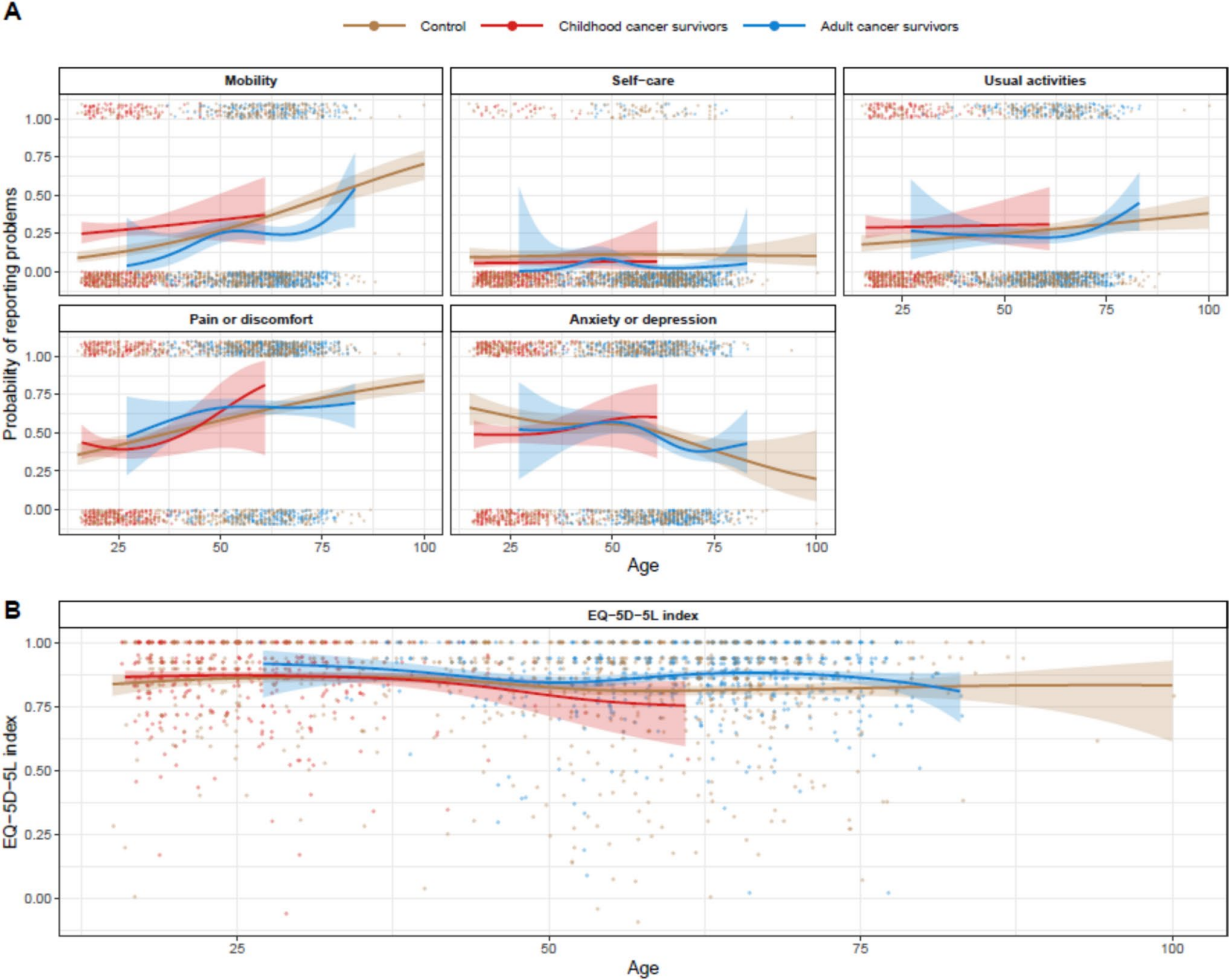
Comparisons between cancer survivors and controls across **A**) HRQoL domains and **B**) overall

On average 51.5% of childhood cancer survivors and 54.9% of adult-onset cancer survivors reported meaningful reductions in overall HRQoL compared to perfect health (i.e. those with an index < 0.92; Fig. 1). Overall HRQoL, as indicated by average EQ-5D-5L index values, did not significantly differ between childhood cancer survivors and age-matched controls (*p* = *0.58, *Fig. 2). However, when each domain of HRQoL was considered, childhood cancer survivors were significantly more likely than age-matched controls to report problems with mobility (*p* < *0.001*) and performing usual activities (*p* = *0.028*) but were less likely to report problems with anxiety or depression (*p* = *0.043,* Fig. 2). We did not find evidence that the magnitude of these differences changed with age at the time of study participation (mobility *p* = *0.11*; usual activities *p* = *0.51*; anxiety or depression *p* = *0.14*). There was no significant difference between groups in the likelihood of reporting problems with self-care (*p* = *0.25*) or pain or discomfort (*p* = *0.39*). Supplementary Table 1 details HRQoL distributions across EQ-5D-5L domains for childhood cancer survivors, by diagnosis.

Adult cancer survivors reported significantly better overall HRQoL than age-matched controls, based on average EQ-5D-5L index values (*p* < *0.001*, Fig. 2). The *magnitude* of this difference between groups did not vary significantly across ages (*p* = *0.066*). Across specific HRQoL domains, adult cancer survivors were less likely to report problems with mobility (*p* < *0.001*) and self-care (p < *0.001*) than age-matched controls, although no differences were observed across other domains.

### Factors associated with childhood cancer survivors’ HRQoL

**Fig. 3:**
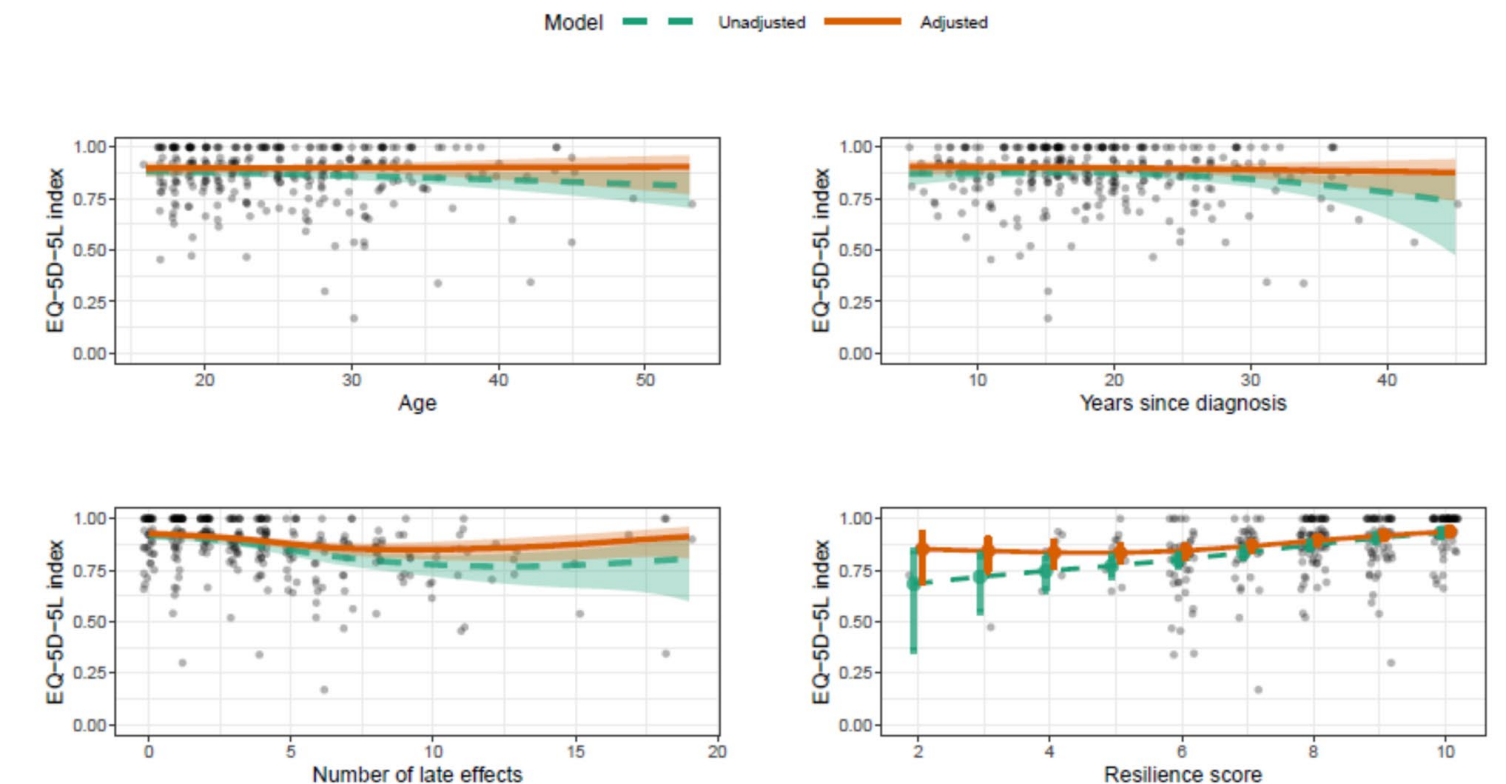
Factors associated with childhood cancer survivors’ HRQoL

The factors listed in Table 2 were included in a multivariable analysis of 288 childhood cancer survivors with non-missing values for these factors. After accounting for other factors, childhood cancer survivors with fewer health issues (*p* < *0.001*), higher resilience scores (*p* < *0.001*) and those without a history of smoking (relative deficit 0.67; 95% CI: 0.50, 0.90; *p* = *0.002*) reported greater HRQoL (Fig. 3). Cancer diagnosis and treatment received were also strongly associated with HRQoL scores (*p* < *0.001*), such that survivors of childhood leukaemia reported significantly poorer HRQoL compared with survivors with other types of cancer diagnoses (relative deficit 2.20; 95% CI: 1.42, 3.40). Among leukaemia survivors, those who had undergone bone marrow transplantation reported better HRQoL compared to those who did not receive this treatment (relative deficit 0.62; 95% CI: 0.40, 0.94). There was some suggestion that lymphoma survivors who had received radiotherapy tended to report poorer HRQoL than those who did not, although this was not significant (relative deficit 1.88; 95% CI: 0.96, 3.67). No other demographic (e.g. sex) or clinical (e.g. time since diagnosis) factors were significantly associated with childhood cancer survivors’ HRQoL.

**Table 2:** Factors associated with HRQoL entered in univariable and multivariable analyses

	Univariable			Multivariable		
	Rel. Def	95%CI	*p*-value	Rel. Def	95%CI	*p*-value
MODEL 1: Factors associated with HRQoL in Childhood cancer survivors						
Female gender (vs male)	1.26	0.99, 1.60	0.074	1.15	0.91, 1.46	0.471
Regional/remote (vs metro)	1.01	0.77, 1.34	0.916	0.99	0.76, 1.29	0.964
Tertiary education (vs secondary)	0.98	0.78, 1.24	0.887	1.11	0.87, 1.43	0.247
Employed (vs unemployed)	0.75	0.59, 0.96	**0.019**	0.93	0.72, 1.20	0.544
Currently married (vs not married)	0.90	0.68, 1.19	0.457	0.93	0.68, 1.26	0.912
Ever smoked (vs never smoked)	1.34	0.99, 1.83	0.086	1.49	1.11, 2.01	**0.002**
Physical activity (vs sedentary)			**0.011**			0.197
Insufficient	0.62	0.46, 0.85		0.77	0.58, 1.03	
Sufficient	0.61	0.44, 0.86		0.87	0.62, 1.20	
Diagnosis + treatment^a^			**0.017**			** < 0.001**
Diagnosis (vs other cancer)						
Lymphoma	0.96	0.59, 1.57		1.05	0.67, 1.67	
Leukaemia	1.97	1.21, 3.23		2.20	1.42, 3.40	
Brain cancer	1.02	0.52, 1.98		0.83	0.46, 1.50	
Surgery (vs no/unknown)^b^						
Lymphoma patients	1.10	0.53, 2.26		1.06	0.55, 2.03	
Radiotherapy (vs no/unknown)^b^						
Leukaemia patients	1.11	0.77, 1.60		0.87	0.60, 1.25	
Other cancer patients	1.21	0.76, 1.91		1.02	0.67, 1.54	
BMT (vs no/unknown)^b^						
Leukaemia patients	0.58	0.36, 0.94		0.62	0.40, 0.94	
Age^c^	-	-	0.246	-	-	0.899
Time since diagnosis^c^	-	-	0.076	-	-	0.338
Total number of late effects^c^	-	-	** < 0.001**	-	-	** < 0.001**
Resilience ^c^	-	-	** < 0.001**	-	-	** < 0.001**
	**UNIVARIABLE**			**MULTIVARIABLE**		
	**Rel. Def**	**95%CI**	**p-value**	**Rel. Def**	**95%CI**	**p-value**
MODEL 2: Factors associated with HRQoL in Adult-onset cancer survivors						
Female gender (vs male)	0.97	0.74, 1.25	0.783	0.84	0.63, 1.13	0.253
Regional/remote (vs metro)	1.09	0.96, 1.25	0.192	0.98	0.86, 1.13	0.757
Tertiary education (vs secondary)	0.75	0.64, 0.87	** < 0.001**	0.79	0.67, 0.92	**0.006**
Employed (vs unemployed)	1.00	0.89, 1.13	0.990	0.95	0.81, 1.12	0.482
Currently married (vs not married)	0.82	0.73, 0.94	**0.003**	0.90	0.79, 1.03	0.187
Ever smoked (vs never smoked)	1.09	0.95, 1.23	0.223	1.10	0.96, 1.26	0.138
Physical activity (vs sedentary)			** < 0.001**			** < 0.001**
Insufficient	0.71	0.60, 0.84		0.79	0.66, 0.94	
Sufficient	0.52	0.45, 0.61		0.66	0.56, 0.78	
Diagnosis + treatment^a^			0.067			0.836
Breast cancer (vs other cancer)	0.93	0.81, 1.08		0.98	0.84, 1.15	
Chemotherapy (vs no/unknown)^b^						
Breast cancer patients	1.23	1.05, 1.44		1.01	0.86, 1.19	
Other cancer patients	0.90	0.70, 1.14		1.04	0.81, 1.34	
Radiotherapy (vs no/unknown)^b^						
Breast cancer patients	1.04	0.88, 1.24		1.07	0.90, 1.27	
Other cancer patients	1.26	1.01, 1.58		1.06	0.84, 1.34	
Age^d^	-	-	** < 0.001**	-	-	**0.013**^e^
Time since diagnosis^d^	-	-	0.275	-	-	0.179^f^
Age x Time since diagnosis interaction^d^	-	-	** < 0.001**	-	-	0.160
Total number of late effects^d^	-	-	** < 0.001**	-	-	** < 0.001**
Resilience^d^	-	-	** < 0.001**	-	-	** < 0.001**

Abbreviations: Rel. Def., relative deficit; CI, confidence interval; ^**a**^ Interaction effect (p-value is a combined test of main effects + interaction; marginal estimates of diagnosis are averaged over treatments); ^**b**^ Only treatment comparisons for diagnoses with minimum 20 patients in each group are shown; ^**c**^ See Fig. 3 for fitted associations; ^**d**^ See Fig. 4 for fitted associations; ^e^ Age adjusted for time since diagnosis; ^**f**^ Time since diagnosis adjusted for age

### Factors associated with adult cancer survivors’ HRQoL

**Fig. 4:**
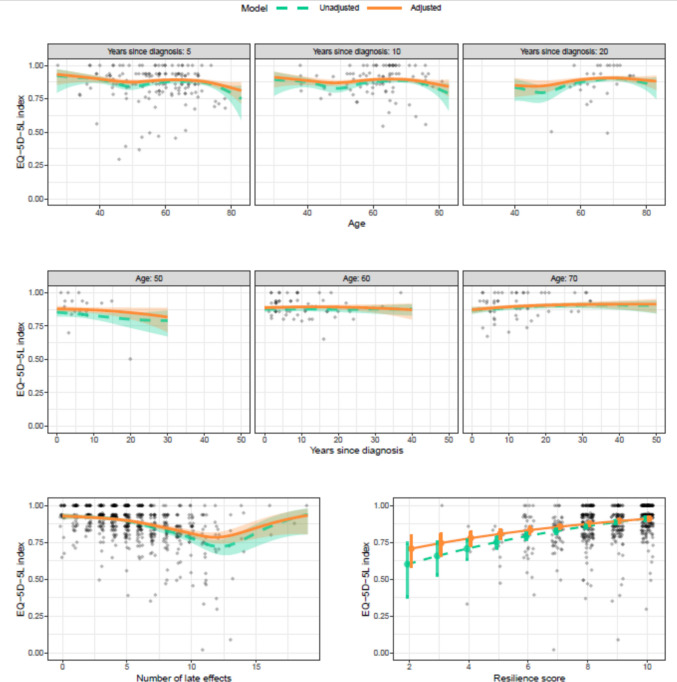
Factors associated with adult cancer survivors’ HRQoL

The factors listed in Table 2 were included in a multivariable analysis of 606 adult cancer survivors with non-missing values for these factors. After accounting for other factors, adult cancer survivors reported HRQoL was significantly better in survivors with tertiary education (relative deficit 0.79; 95% CI: 0.67, 0.92; *p* = *0.006*), those who reported higher resilience scores (*p* < *0.001*), reported a higher total number of late effects (*p* < *0.*001), and those who were physically active compared to sedentary (*p* < *0.001*). There was evidence that HRQoL differed by age (*p* = *0.013*), with adult cancer survivors aged around 50 years old reporting lower HRQoL than otherwise similar survivors of different ages (Fig. 4). There was no evidence that this pattern changed with time since diagnosis (*p* = *0.16*), or that time since diagnosis was itself an important factor (*p* = *0.18*). No other demographic (e.g. sex) or clinical (e.g. time since diagnosis) factors were significantly associated with adult cancer survivors’ HRQoL.

## Discussion

The purpose of this study was to describe the health-related quality of life (HRQoL) of Australian cancer survivors across the lifespan compared to those with no history of cancer and to identify factors associated with survivor HRQoL. For survivors of childhood cancer, overall HRQoL was no different to age-matched controls. On average, 51.5% of childhood cancer survivors reported overall HRQoL less than perfect health. When specific domains of HRQoL were considered, childhood cancer survivors were more likely to report difficulties with mobility and performing usual activities, and less likely to report difficulties with anxiety and depression compared to controls. Survivors of adult-onset cancer reported better overall HRQoL than age-matched controls and were less likely to report difficulties in mobility and self-care. However, on average, 54.9% of survivors of adult-onset cancer reported meaningful reductions in overall HRQoL compared to perfect health (i.e., those with an index < 0.92).

Although we did not observe any association between time since diagnosis and HRQoL among survivors of childhood cancer and adult-onset cancer, longitudinal studies have shown HRQoL declines significantly post-treatment and then stabilizes over time the further away from diagnosis [6, 9, 10]. These patterns in HRQoL in the post-treatment and survivorship phase may reflect adjustment to a “new normal” after cancer, and a revaluing of one’s’ physical, role, social and psychological functioning during survivorship relative to functioning while undergoing cancer treatment [54]. Across our sample of survivors of childhood and adult-onset cancer we found some domains of HRQoL were negatively impacted while others were generally on par with age-matched peers. These results highlight the need to consider individual-level factors that may be associated with better HRQoL during survivorship.

In our study, HRQoL was poorer in lymphoma and leukaemia survivors overall compared to controls. These findings echo existing literature [2, 55], and further highlight the focus on the well-documented medical and treatment factors that have been identified to date. In the post-treatment phase, our findings reinforce that childhood cancer survivors who reported a higher number of cancer-related health issues also reported poorer HRQoL [3, 33]. Managing these cancer-related late effects is a crucial factor in optimising HRQoL for the growing population of cancer survivors, yet further complicated by certain symptom clusters that together result in a higher overall burden and impact on HRQoL [56]. For example, fatigue, sleep disturbance, and chronic pain commonly co-occur in childhood cancer survivors and can interact to exacerbate functional limitations, thereby compounding the overall impact on HRQoL [57]. Many cancer-related symptoms and health problems may co-occur and are often interrelated, necessitating holistic psychosocial support for cancer survivors that may more effectively improve HRQoL [58].

Among our adult-onset survivors however we observed the opposite, that greater late effect burden was associated with better HRQoL. This may reflect sample characteristics linked to better wellbeing (e.g., higher education, female sex), the resilience to overcome multi-morbidity, or engagement in survivorship care that support functioning even in the presence of multiple conditions. Recent studies similarly highlight the roles of resilience, adaptive coping, and high-quality survivorship care in sustaining HRQoL among adult cancer survivors with substantial symptom or morbidity burdens and may be an important target for clinical intervention [59]. The unexpectedly higher overall HRQoL observed among adult-onset cancer survivors may be attributable to selection bias toward higher-functioning individuals in online recruitment, shorter time since diagnosis with greater opportunity for adaptation, or the influence of higher resilience within this sample [59, 60]. On the other hand, childhood cancer survivors reported lower rates of anxiety/depression compared with controls which may reflect survivorship bias, routine psychosocial screening and early intervention in pediatric follow-up care, or post-traumatic growth facilitated by strong family and clinical support during treatment [61, 62].

Resilience was also strongly associated with HRQoL across both childhood and adult-onset cancer survivors in our study. Resilience has been described as a dynamic process of coping and adapting to stressful circumstances such as the experience of cancer and treatment [63]. Previous studies have demonstrated a link between resilience and adaption to the experience of cancer in patients currently undergoing treatment [64–66], and patients’ family members or caregivers [67–69]. In several studies, older survivors of adult-onset cancers reported less distress and greater adaptability than younger survivors, potentially due to the relative infrequency of cancer as a younger adult and thus greater likelihood of its disruptive nature and greater difficulty adjusting [67, 68, 70, 71]. Whilst promoting psychological resilience appears to be a promising strategy for improving HRQoL, few evidence-based interventions exist tailored to the unique psychosocial needs of childhood, adolescent, and (young) adult cancer survivors [72, 73].

Childhood cancer survivors who had a history of smoking had poorer HRQoL than controls. The effects of smoking on health are well-documented and are strongly linked to poorer health outcomes in general and cancer populations [74–76]. With a recent rise in uptake of nicotine products among young Australians [77], these findings highlight survivors as particularly vulnerable to negative impacts of smoking behaviours on HRQoL. Alongside public health strategies to reduce smoking, and more recently global efforts to reduce e-cigarette use or ‘vaping’, survivors will benefit from education and/or support to minimise smoking uptake/continuation incorporated into their regular survivorship care [78].

Among adult-onset cancer survivors in our study, engagement in some level of physical activity was associated with better HRQoL than controls, regardless of whether they met the recommended guidelines. Less than 40% of Australian cancer survivors meet the government recommended requirements for weekly physical activity [79], despite the known benefits. With a significantly greater proportion of childhood cancer survivors in our study experiencing problems with mobility, this highlights a key at-risk group for poor HRQoL. Modifying lifestyle behaviours in general, including sleep, alcohol/substance use, or weight management, is critical for preventing several health problems that may impact HRQoL. Yet, some lifestyle changes such as discouraging smoking and increasing physical activity levels can be challenging for cancer survivors, particularly among those who already report reduced quality of life.

Strengths of this study include the large cohort sample size, the use of validated patient-reported outcomes, inclusion of survivors across all ages at diagnosis, and comparison to people without a history of cancer. Although we recruited childhood cancer survivors from all 11 tertiary centres across Australia and New Zealand enhancing representativeness, the sample may still reflect a somewhat healthier or more engaged subset of childhood cancer survivors. Our study is potentially subject to selection bias since approximately fewer than 25% of eligible childhood cancer survivors and 1.5% of adult cancer survivors participated, which may reflect a relatively healthier and more engaged subset of survivors, limiting generalisability to the broader survivor population. Despite our sample size, females and breast cancer survivors who received surgery accounted for the majority of the adult-onset survivor group, which limits the generalisability of our results to males and other cancer diagnoses and treatment modalities. Regression findings should be interpreted cautiously as adjusted contrasts rather than independent effects. This is important given the prevalence of other diagnoses and treatment modalities experienced by adult survivors. For example, over 95% of men in Australia diagnosed with testicular and prostate cancer survive at least 5 years post-diagnosis and prostate cancer was the most common diagnosis among men in 2022 [80]. Additionally, our results relating to self-care and mobility may be attributed to the high level of education and predominately female adult survivor sample, such that health literacy, proactive self-management and engagement in help seeking may be more likely among educated females and support greater functional and mobility outcomes [81, 82]. Future research ought to clarify HRQoL outcomes and associated factors among adult male survivors and survivors with poorer health behaviours, lower education, or less access to support/resources. Future studies with larger adolescent and young adult cohorts (15–39 years) should also stratify analyses to elucidate these distinct survivorship trajectories which may have been masked in our study by grouping them with adult-onset survivors.

We also lacked non-respondent data for survivors of adult-onset cancer, making it difficult to ascertain how representative our sample is of the broader population. Our questionnaire was only available to English speaking participants and the sample of participating survivors was also highly educated (52% of childhood and 83% adult cancer survivors reported a tertiary education). Participants may have reported higher HRQoL than among the broader population of cancer survivors, given the documented association between education and HRQoL [27, 83]. In our study, we did not control for factors including educational attainment, income, employment or other factors which may be important socioeconomic indicators driving HRQoL, and potentially accounting for some of the differences observed between cancer survivors and controls. We also did not collect data on specific treatment protocols or intensity, nor did we evaluate the potential differences in cancer diagnoses on physical activity which may be valuable areas for future research. While our findings align with survivorship patterns reported in other high-income countries, differences in healthcare systems, access to survivorship care, and population demographics mean that generalisability to other developed settings may be limited.

Regarding clinical implications, our findings highlight the need for tailored survivorship care approaches that address cancer survivors’ unique vulnerabilities that may impact HRQoL. For example, particular attention should be given to survivors with lower educational attainment or those experiencing multiple cancer-related health problems that might indicate higher symptom burden and consequently poorer HRQoL [33, 83]. During diagnosis and treatment, interventions are needed to minimise the impact of cancer on young people academically, given they are more likely to have poorer educational outcomes and tertiary education attainment [84]. The early survivorship phase is an important opportunity to also prioritise survivor education and thus preparedness through comprehensive information and support. This may help to foster survivors’ resilience and may help to mitigate psychological outcomes (e.g. fear of recurrence) which are key psychosocial factors associated with HRQoL [33]. In the longer term, routine screening and interventions targeting modifiable lifestyle risks like smoking, physical inactivity, and poor diet quality are warranted, although more evidence is needed as to the most effective means of supporting such lifestyle changes in cancer survivors [85–87]. Care models that more holistically address the management of chronic conditions, health behaviour change, and psychosocial needs are recommended to maximise HRQoL outcomes. Collectively, this calls for risk-stratified, holistic survivorship care pathways that proactively identify and allow for intervention of the multifaceted determinants of HRQoL across the cancer survivorship continuum [4].

In this study we sought to describe HRQoL in childhood and adult-onset cancer survivors. Compared to healthy controls, HRQoL was similar among childhood cancer survivors and better in survivors of a cancer diagnosed in adulthood. Several factors were significantly associated with HRQoL in childhood and adult-onset survivors including various modifiable factors which could inform the design and delivery of survivorship care. These data underscore the need for integrated, holistic models of care that seamlessly bridge oncology and primary care services. By proactively managing comorbidities and late effects, such collaborative approaches could not only optimize physical health outcomes but also alleviate the compounding effects on survivors' functional status and quality of life.

## Supplementary Information

Below is the link to the electronic supplementary material.

ESM 1 (DOCX 18.3 KB)

## Data Availability

The data that support the findings of this study are not publicly available due to privacy or ethical restrictions, and the full dataset is not able to be released due to ethical restrictions. Requests may be made to the authors.
