# Supplementary material for: Factors associated with health-related quality of life in survivors of childhood-onset and adult-onset cancer compared to controls
Source: Support Care Cancer. 2026 May 14;34(6):539. doi: 10.1007/s00520-026-10775-y (PMC13176070; doi:10.1007/s00520-026-10775-y)
Supplement: Supplementary file 1 — (DOCX 18.3 KB) [file 520_2026_10775_MOESM1_ESM.docx]

**Supplementary Table 1**. Health-related quality of life (EQ-5D-5L) domain distributions for childhood cancer survivors, by diagnosis

| Domain | Response | Lymphoma (n=57) | Leukaemia (n=174) | Brain cancer (n=53) | Other (n=117) | (Unknown) (n = 2) |
| --- | --- | --- | --- | --- | --- | --- |
| Mobility | No problems | 43 | 135 | 31 | 83 | 1 |
|  | Slight problems | 12 | 27 | 17 | 23 | 1 |
|  | Moderate problems | 1 | 9 | 3 | 5 | 0 |
|  | Severe problems | 1 | 3 | 1 | 4 | 0 |
|  | I am unable to | 0 | 0 | 1 | 1 | 0 |
|  | (No response) | 0 | 0 | 0 | 1 | 0 |
| Self care | No problems | 57 | 163 | 50 | 107 | 2 |
|  | Slight problems | 0 | 8 | 1 | 6 | 0 |
|  | Moderate problems | 0 | 2 | 2 | 3 | 0 |
|  | Severe problems | 0 | 1 | 0 | 0 | 0 |
|  | I am unable to | 0 | 0 | 0 | 0 | 0 |
|  | (No response) | 0 | 0 | 0 | 1 | 0 |
| Usual activities | No problems | 42 | 130 | 32 | 81 | 0 |
|  | Slight problems | 11 | 29 | 14 | 27 | 1 |
|  | Moderate problems | 4 | 10 | 6 | 5 | 1 |
|  | Severe problems | 0 | 4 | 1 | 1 | 0 |
|  | I am unable to | 0 | 1 | 0 | 1 | 0 |
|  | (No response) | 0 | 0 | 0 | 2 | 0 |
| Pain | No pain | 34 | 104 | 30 | 66 | 1 |
|  | Slight pain | 19 | 42 | 16 | 30 | 1 |
|  | Moderate pain | 4 | 21 | 5 | 14 | 0 |
|  | Severe pain | 0 | 6 | 2 | 4 | 0 |
|  | Extreme pain | 0 | 1 | 0 | 2 | 0 |
|  | (No response) | 0 | 0 | 0 | 1 | 0 |
| Anxiety / depression | Not anxious or depressed | 27 | 86 | 27 | 63 | 0 |
|  | Slightly anxious or depressed | 21 | 43 | 18 | 30 | 0 |
|  | Moderately anxious or depressed | 7 | 29 | 7 | 16 | 1 |
|  | Severely anxious or depressed | 0 | 10 | 1 | 6 | 1 |
|  | Extremely anxious or depressed | 2 | 4 | 0 | 1 | 0 |
|  | (No response) | 0 | 2 | 0 | 1 | 0 |
